# Anti-Photoaging and Anti-Melanogenesis Effects of Fucoidan Isolated from *Hizikia fusiforme* and Its Underlying Mechanisms

**DOI:** 10.3390/md18080427

**Published:** 2020-08-15

**Authors:** Lei Wang, Jae-Young Oh, Young-Sang Kim, Hyo-Geun Lee, Jung-Suck Lee, You-Jin Jeon

**Affiliations:** 1 Department of Marine Life Sciences, Jeju National University, Jeju Self-Governing Province, Jeju 63243, Korea; comeonleiwang@163.com (L.W.); ojy0724@naver.com (J.-Y.O.); medieval032@naver.com (Y.-S.K.); hond0502@hanmail.net (H.-G.L.); 2 Marine Science Institute, Jeju National University, Jeju Self-Governing Province, Jeju 63333, Korea; 3 Research Center for Industrial Development of Seafood, Gyeongsang National University, Tongyeong 53064, Korea

**Keywords:** fucoidan, skin health, anti-melanogenesis effect, anti-photoaging effect

## Abstract

Previous studies suggested that fucoidan with a molecular weight of 102.67 kDa, isolated from *Hizikia fusiforme*, possesses strong antioxidant activity. To explore the cosmeceutical potential of fucoidan, its anti-photoaging and anti-melanogenesis effects were evaluated in the present study. The anti-photoaging effect was investigated in ultraviolet (UV) B-irradiated human keratinocytes (HaCaT cells), where fucoidan effectively reduced the intracellular reactive oxygen species level and improved the viability of the UVB-irradiated cells without any cytotoxic effects. Moreover, fucoidan significantly decreased UVB-induced apoptosis in HaCaT cells by regulating the protein expression of Bax, Bcl-xL, PARP, and Caspase-3 in HaCaT cells in a concentration-dependent manner. The anti-melanogenesis effect of fucoidan was evaluated in B16F10 melanoma cells that had been stimulated with alpha-melanocyte-stimulating hormone (α-MSH), and fucoidan treatment remarkably inhibited melanin synthesis in α-MSH-stimulated B16F10 cells. Further studies indicated that fucoidan significantly suppressed the expression of tyrosinase and tyrosinase-related protein-1 and -2 (TRP-1 and-2) in B16F10 cells by down-regulating microphthalmia-associated transcription factor (MITF) through regulation of the ERK–MAPK (extracellular signal regulated kinase-mitogen activated protein kinase) pathway. Taken together, these results suggest that fucoidan isolated from *H. fusiforme* possesses strong anti-photoaging and anti-melanogenesis activities and can be used as an ingredient in the pharmaceutical and cosmeceutical industries.

## 1. Introduction

Skin is the largest organ in the human body. As a barrier, skin protects the body against external stimuli, such as particulate matter, chemicals, and ultraviolet (UV) irradiation [1,2]. The UV irradiation from sunlight is considered to be the primary environmental factor that causes skin damage, a process referred to as photoaging [3]. It leads to sunburn, erythema, and skin aging, as well as skin cancer [4]. UV is divided into three main bands according to the wavelength: the 100–280 nm band (designated as UVC), 280–320 nm band (designated as UVB), and 320–400 nm band (designated as UVA) [2]. Among these three bands, UVB is the key factor during skin extrinsic aging [5]. Thus, there has been more investigative attention paid to the mechanisms of UVB-induced skin photoaging.

Melanogenesis is the physiological process that results in the production of melanin, a pigment that contributes to skin and hair color. Melanin also plays an important role in the prevention of UV-induced skin damage [6,7,8]. However, an abnormally excessive production and accumulation of melanin could cause pathological and cosmetic problems. Melanin overproduction can be stimulated by various factors such as the abnormal release of alpha-melanocyte-stimulating hormone (α-MSH), inflammation, and UV irradiation [9,10]. Asian countries, such as China, Korea, and Japan, have a long history of considering fair skin tone as being one of the main criteria of personal beauty [11,12]. In addition, an increasing number of Asian women aspire to obtain a fairer skin complexion [13]. Therefore, a safe and effective agent that inhibits melanogenesis without causing side effects is desired.

Because natural products have high physiological effects and low or no toxicity, recently, ingredients from natural sources have tended to dominate the cosmetic market. Seaweeds are rich in natural bioactive compounds such as polysaccharides, pigments, lipids, and peptides, which possess extensive health benefits to humans [14,15,16]. In particular, the ingredients from edible or cultivable seaweeds have caught the attention of the nutraceutical, pharmaceutical, and cosmeceutical industries [17,18]. Various studies have reported the potential of fucoidan in cosmetics [19,20,21]. Katsube et al. have investigated the hyaluronidase inhibitory activity of a fucoidan isolated from *Underia pinnatifida* [19]. The results indicated that the fucoidan isolated from *U. pinnatifida* significantly and concentration-dependently inhibited hyaluronidase activity and suggested that it may be a potential candidate to suppress skin inflammation through inhibiting hyaluronidase activity [19]. Pozharitskaya et al. have investigated the pharmacokinetics of fucoidan after topical application to rats [20]. The results indicated no accumulation of fucoidan in plasma was observed after repeated topical applications of 100 mg/kg during five days and supported the rationality of topical application of formulations with fucoidan [20].

*Hizikia fusiforme*, an edible brown seaweed, is one of the most popular seaweeds consumed in China, Korea, and Japan. It is used widely as a food and medicinal ingredient in Asian countries and is cultivated on a vast scale in coastal zones in Asia as an economic seaweed [22,23,24]. Previous reports have suggested that the polysaccharides from *H. fusiforme* possess various bioactivities such as antioxidant, anti-virus, anti-cancer, anti-inflammatory, and anti-diabetic activities [22,23,24,25,26,27,28]. In a previous study, we had isolated a fucoidan from *H. fusiforme* and found that it possessed strong antioxidant activity, which suggested its cosmeceutical potential of the fucoidan [29]. To further explore the fucoidan for cosmeceutical purposes, its anti-photoaging and anti-melanogenesis effects were investigated in the present study.

## 2. Results and Discussion

### 2.1. Anti-Photoaging Effect of Fucoidan

Reactive oxygen species (ROS) play an important role in human health because they are related to various diseases. Abnormal ROS production leads to various adverse effects, including damage to essential macromolecules such as DNA, lipids, and proteins [30,31,32,33,34]. Accumulation of this molecular damage can subsequently cause cell apoptosis, necrosis, and death. UVB irradiation stimulates intracellular ROS production in skin cells and causes photoaging [35]. Various reports have suggested that UVB-induced skin photoaging could be suppressed by polysaccharides isolated from seaweeds [36,37,38]. Thevanayagam et al. investigated the photoprotective effect of the carrageenan isolated from *Eucheuma sp.* and found the carrageenan effectively reduced the intracellular ROS level in UVB-irradiated HaCaT cells and increased the viability of the cells [36]. In our previous study, we investigated the UVB protective effect of the crude sulfated polysaccharides isolated from *H. fusiforme* (HFPS) and found that HFPS effectively protected HaCaT cells against UVB-induced photoaging [39]. However, the photoprotective effect of the purified fucoidan and its potential mechanism of action have not been investigated so far. Therefore, in the present study, we evaluated the effect of fucoidan on UVB-induced photoaging and its photoprotective mechanism.

**Figure 1:**
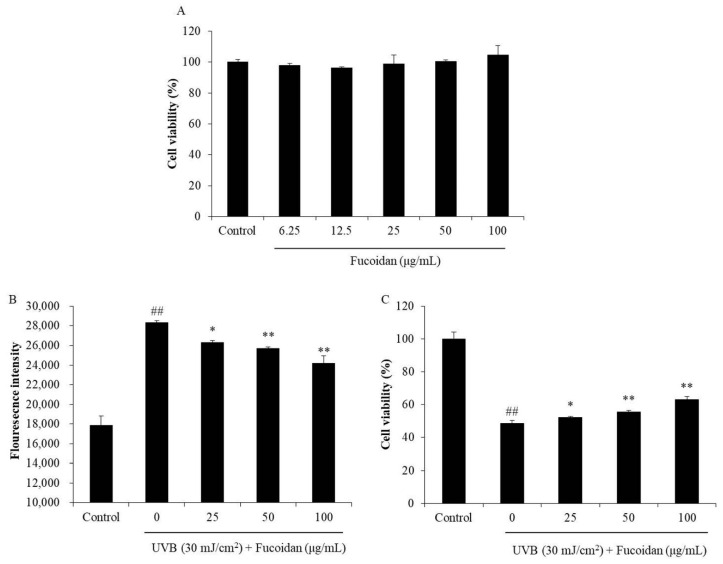
Protective effect of fucoidan against UVB (ultraviolet B)-induced HaCaT cell damage. (**A**) Cytotoxicity of fucoidan in HaCaT cells; (**B**) intracellular reactive oxygen species (ROS) level in UVB-irradiated HaCaT cells; (**C**) viability of UVB-irradiated HaCaT cells. Cell viability was measured with the MTT assay, and the intracellular ROS levels were determined with the DCF-DA assay. All experiments were conducted in triplicate, and the data are expressed as the mean ± SE. * *p* < 0.05, ** *p* < 0.01 when compared with the UVB-irradiated group and ^##^ *p* < 0.01 when compared with the control group.

As shown in Figure 1A, the percentages of viable HaCaT cells treated with different concentrations of fucoidan (6.25–100 μg/mL) were all higher than 95%. It means that fucoidan below the concentration of 100 μg/mL is non-toxic to cells. Thus, 100 μg/mL was applied as the maximum concentration in the further experiments. The photoprotective effect of fucoidan was investigated by evaluating its intracellular ROS-scavenging and cytoprotective effects in UVB-irradiated HaCaT cells. As Figure 1B shows, UVB significantly induced intracellular ROS generation in HaCaT cells, but the ROS level was significantly reduced by fucoidan treatment in a concentration-dependent manner (Figure 1B). In addition, the viability of the UVB-irradiated HaCaT cells was significantly decreased compared with that of their non-UVB-irradiated cells (Figure 1C). However, fucoidan effectively increased the viability of the UVB-irradiated HaCaT cells in a concentration-dependent manner (Figure 1C). These results demonstrated that fucoidan could effectively protect HaCaT cells against UVB-induced cell death and possibly achieved this by scavenging intracellular ROS. Su et al. have evaluated the photoprotective effect of fucoidan (LJSF4) isolated from *Saccharina japonica* in HaCaT cells [40]. The results indicated LJSF4 contains 56.55% carbohydrate and 30.72% sulfate contents, and it increased the viability of UVB-irradiated HaCaT cells by 16.13% at the concentration of 100 μg/mL [40]. Compared with the present results, LJSF4 possesses a slightly stronger activity than the fucoidan isolated from *H. fusiforme*, possibly owing to its higher sulfate content.

**Figure 2:**
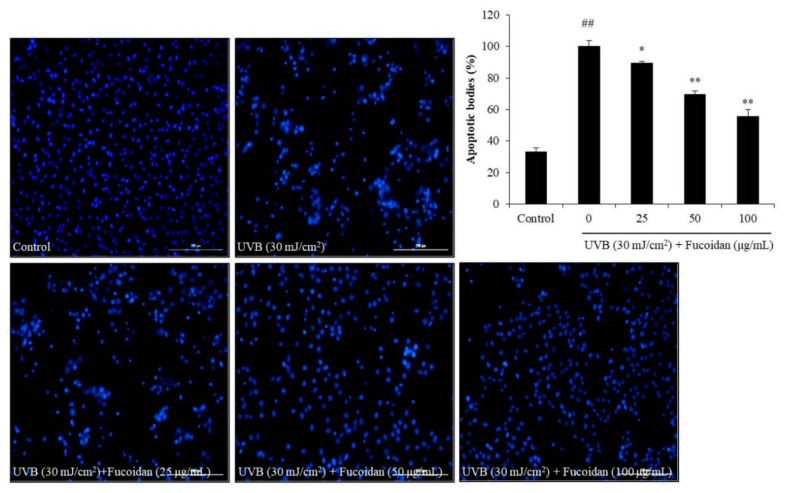
Fucoidan suppresses UVB-induced apoptosis in HaCaT cells. The Hoechst-stained cells were observed under a fluorescence microscope, and the relative levels of apoptosis were measured using Image J software. The data are expressed as the mean ± SE (*n* = 3). * *p* < 0.05, ** *p* < 0.01 when compared with the UVB-irradiated group and ^##^ *p* < 0.01 when compared with the control group.

**Figure 3:**
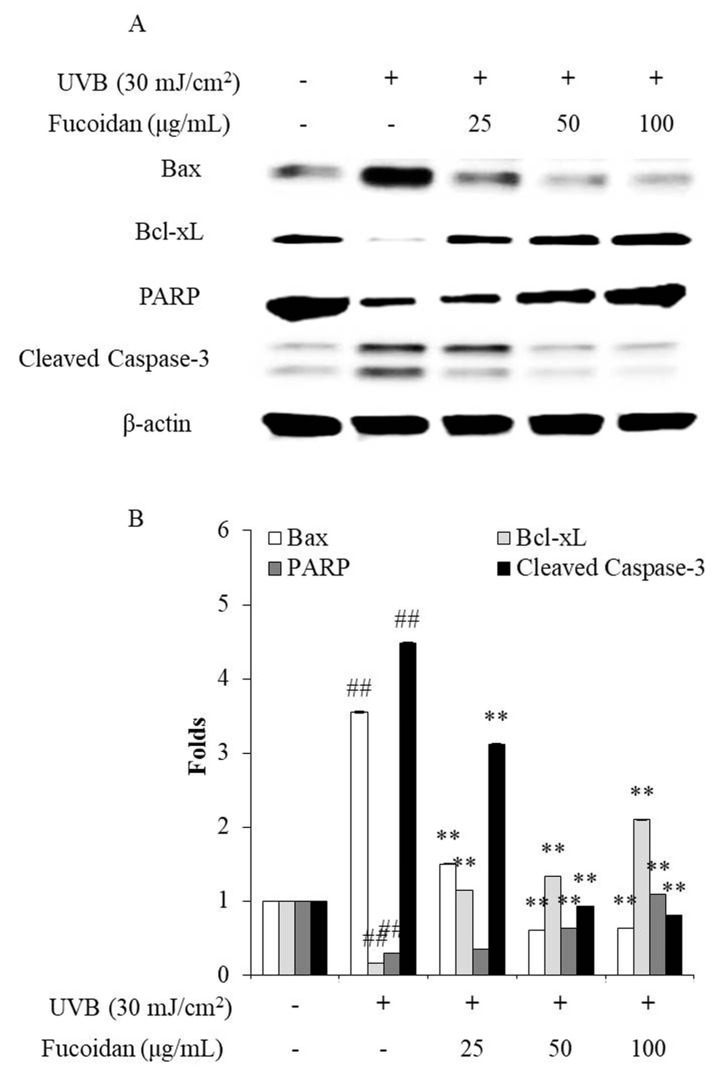
Effects of fucoidan on the Bax, Bcl-xL, PARP (Poly (ADP-ribose) polymerase), and cleaved Caspase-3 expression levels in UVB-irradiated HaCaT cells. (**A**) Fucoidan regulated the Bax, Bcl-xL, PARP, and cleaved Caspase-3 levels in UVB-irradiated HaCaT cells; (**B**) relative amounts of Bax, Bcl-xL, PARP, and cleaved Caspase-3 compared with that of β-actin. The data are expressed as the mean ± SE (*n* = 3). ** *p* < 0.01 when compared with the UVB-irradiated group and ^##^ *p* < 0.01 when compared with the control group.

Cell death can occur through three routes: autophagy, necrosis, and apoptosis. Apoptosis is an intrinsic cellular suicidal mechanism, which is regulated by a complex network of signaling pathways, such as Caspase, Bax, Bcl-xL, and PARP pathway [41,42,43,44]. To further investigate the photoprotective mechanism of fucoidan, the apoptotic bodies and the expression of apoptosis-related proteins in UVB-irradiated HaCaT cells were measured. The apoptotic body formation was measured via Hoechst 33342 staining. As shown in Figure 2, UVB irradiation significantly induced apoptotic body formation in HaCaT cells, whereas the amounts of apoptotic bodies of fucoidan-treated HaCaT cells were remarkably decreased in a concentration-dependent manner (Figure 2). Additionally, UVB irradiation elevated the expression of the apoptotic proteins (Bax and cleaved Caspase-3) and reduced the anti-apoptosis proteins (Bcl-xL and PARP) (Figure 3). However, fucoidan not only reduced the cleaved Caspase-3 and Bax levels but also improved the Bcl-xL and PARP levels in UVB-irradiated HaCaT cells (Figure 3). Both effects were concentration dependent. These results indicate that fucoidan has a potent effect in protecting HaCaT cells against UVB-induced apoptosis through regulation of apoptosis-related signaling pathways. Taken together, these results demonstrate that fucoidan possesses a strong capability to protect cells against UVB-induced photoaging and likely achieves this by reducing cell death through intracellular ROS scavenging to regulate the apoptosis-related signaling pathways.

### 2.2. Anti-Melanogenesis Effect of Fucoidan

**Figure 4:**
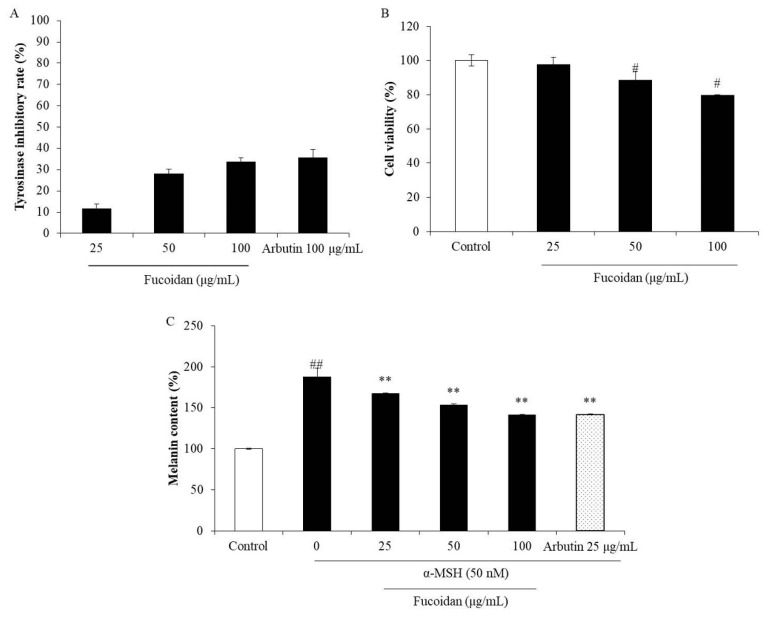
Inhibitory effects of fucoidan on tyrosinase and melanin biosynthesis. (**A**) Inhibitory effect of fucoidan on tyrosinase activity; (**B**) cytotoxicity of fucoidan in B16F10 cells; (**C**) inhibitory effect of fucoidan on melanin biosynthesis in α-MSH (alpha-melanocyte-stimulating hormone)-stimulated B16F10 cells. The data are expressed as the mean ± SE (*n* = 3). ** *p* < 0.01 when compared with the α-MSH-stimulated group and ^#^ *p* < 0.05 and ^##^ *p* < 0.01 when compared with the control group.

Abnormal melanogenesis causes skin pigment disorders, such as freckles and erythema [45]. Because tyrosinase is the key enzyme in the process of melanin biosynthesis, a tyrosinase inhibitor may be a potential candidate for inhibiting or reducing melanin biosynthesis. Therefore, the effect of fucoidan on mushroom tyrosinase was investigated in the present study. As shown in Figure 4A, the inhibitory rates of fucoidan on tyrosinase activity were 11.60%, 28.11%, and 33.62% at the concentrations of 25, 50, and 100 μg/mL, respectively. This inhibitory effect of fucoidan at the high concentration (100 μg/mL) is close that of to the well-known skin-whitening compound arbutin (35.64%). These results indicate that fucoidan possesses strong tyrosinase-inhibiting activity and suggest its potential in inhibiting melanogenesis. To further investigate the effect of fucoidan on melanogenesis, melanin biosynthesis was evaluated in α-MSH-induced B16F10 cells treated with various concentrations of the fucoidan. The melanin content in non-treated α-MSH-stimulated B16F10 cells was significantly increased but was decreased by fucoidan treatment in a concentration-dependent manner (Figure 4C). However, fucoidan showed slight cytotoxicity on B16F10 cells (Figure 4B). According to these results, 25 μg/mL was determined as the safe concentration to use for the further investigations of the anti-melanogenesis mechanism.

**Figure 5:**
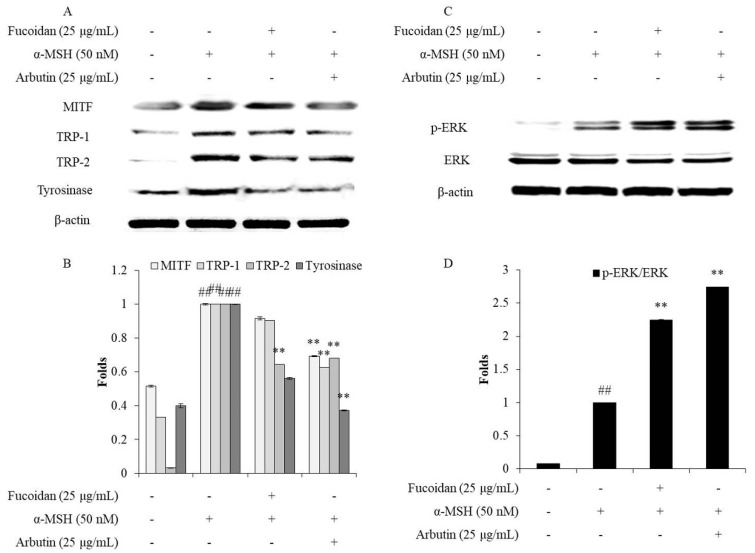
Fucoidan suppresses MITF (microphthalmia-associated transcription factor), tyrosinase, TRP-1 (tyrosinase-related protein-1) and TRP-2 expression, and phosphorylates ERK (extracellular signal regulated kinase) in α-MSH-stimulated B16F10 cells. (**A**) Fucoidan down-regulated the MITF, tyrosinase, TRP-1, and TRP-2 levels in α-MSH-stimulated B16F10 cells; (**B**) relative amounts of MITF, tyrosinase, TRP-1, and TRP-2. (**C**) Fucoidan activated ERK in α-MSH-stimulated B16F10 cells; (**D**) relative amount of activated ERK. The relative amounts of MITF, tyrosinase, TRP-1, TRP-2, ERK, and p-ERK were compared with that of β-actin. ** *p* < 0.01 when compared with the α-MSH-stimulated group, and ^##^ *p* < 0.01 when compared with the control group.

In humans, melanin biosynthesis occurs in the melanocytes and is regulated by various proteins such as tyrosinase, TRP-1 (tyrosinase-related protein-1), TRP-2, and MITF (microphthalmia-associated transcription factor) [46]. Therefore, the regulation of the expression of these proteins is a feasible strategy for inhibiting melanogenesis. Both TRP-1 and TRP-2 are important proteins during melanin biosynthesis because they are related to the stability and activity of tyrosinase. Furthermore, the expression of tyrosinase, TRP-1, and TRP-2 is activated by MITF, which is regulated by the MAPK (mitogen activated protein kinase) signaling pathways, including ERK (extracellular signal regulated kinase), JNK (c-Jun N-terminal kinase), and p38 MAPK [13,47]. In particular, the ERK–MAPK signaling pathway, which is considered to be a negative feedback mechanism in melanogenesis, has been widely studied by other researchers [7,9,47]. Thus, to understand the mechanism behind the inhibitory effect of fucoidan on α-MSH-stimulated melanogenesis in B16F10 cells, its effects on the expression of tyrosinase, TRP-1, TRP-2, and MITF, as well as the activation of the ERK–MAPK signaling pathway, were examined. As Figure 5A,B show, α-MSH significantly stimulated the expression of tyrosinase, TRP-1, TRP-2, and MITF in B16F10 cells, but fucoidan effectively reversed the stimulatory effects by reducing the expression of these proteins. In addition, fucoidan remarkably improved the activated ERK–MAPK levels in the α-MSH-stimulated B16F10 cells (Figure 5C, D). These results suggest that fucoidan inhibits α-MSH-stimulated melanin biosynthesis in B16F10 cells by regulating the ERK–MAPK pathway to inhibit MITF and thereby down-regulate the tyrosinase, TRP-1, and TRP-2 levels. Taken together, these results indicate that fucoidan possesses strong inhibitory activity on melanogenesis and would, therefore, be a potential candidate for skin-whitening products.

## 3. Materials and Methods

### 3.1. Reagents and Chemicals

Mushroom tyrosinase, α-MSH, dimethyl sulfoxide, MTT, and 2,7-dichlorofluorescein diacetate (DCFH_2_-DA) were purchased from Sigma Co. (St. Louis, MO, USA). Penicillin/streptomycin (P/S), Dulbecco’s modified Eagle’s medium (DMEM), and fetal bovine serum (FBS) were purchased from Gibco BRL (Life Technologies, Burlington, ON, Canada). Tyrosinase, Bcl-xL, Bax, tyrosinase-related protein-1 and -2 (TRP-1 and -2), PARP, ERK and p-ERK, cleaved Caspase-3, and β-actin antibodies were purchased from Thermo Fisher Scientific (Waltham, MA, USA). Anti-mouse and anti-rabbit IgG antibodies were purchased from Cell Signaling Technology (Beverly, MA, USA). All other chemicals used in this study were analytical grade.

### 3.2. Sample Preparation

The fucoidan from *H. fusiforme* was prepared using the method described in our previous study [28]. In brief, crude sulfated polysaccharides of *H. fusiforme* (HFPS) were obtained by digestion using Celluclast and ethanol precipitation. A carbohydrate rich fraction (HFPS-F4) was purified from HFPS employing a DEAE-cellulose column [28]. HFPS-F4 contains 99.01% of fucoidan that consisted of 71.79% carbohydrate and 27.22% sulfate content and could be thought of as a fucoidan. Fucoidan (HFPS-F4) has a molecular weight of 102.67 kDa and is composed of 79.20% fucose, 0.19% glucose, 2.09% rhamnose, 18.13% mannose, and 0.38% arabinose.

### 3.3. Determination of the Effect of Fucoidan on Photoaging

#### 3.3.1. Maintenance of HaCaT Cells and Application of UVB to HaCaT Cells

Human keratinocytes (HaCaT cells) were purchased from the Korean Cell Line Bank (Seoul, Korea), and maintained in DMEM (10% FBS and 1% P/S), and subcultured every 3 days. For the experiments, the cells were seeded at a density of 1.0 × 10^5^ cells/mL. According to our previous studies, 30 mJ/cm^2^ of UVB caused around 50% cell death of HaCaT cells [48,49,50]. Thus, in the present study, 30 mJ/cm^2^ of UVB was applied to HaCaT cells to stimulate photodamage. UVB irradiation was imposed using a UVB meter (UV Lamp, VL-6LM; Vilber Lourmat, Torcy, France) with a fluorescent bulb emitting 280–320 nm wavelengths with a peak at 313 nm. Cells were exposed to UVB in PBS and subsequently incubated with serum-free DMEM until analysis [48,49,50].

#### 3.3.2. Measurement of the Effect of Fucoidan on UVB-Induced Photodamage in HaCaT Cells

Before measuring the effect of fucoidan on UVB-induced photodamage, its toxicity to HaCaT cells was measured. HaCaT cells were seeded in a 24-well plate and incubated for 24 h. The cells were treated with fucoidan (6.25, 12.5, 25, 50, and 100 µg/mL) for 24 h, following which the viability of the cells was determined by MTT assay according to the method described previously [49,50]. The effect of fucoidan on UVB-induced photodamage was then evaluated by measuring the level of intracellular ROS, apoptotic bodies formation, and the viability of UVB-irradiated HaCaT cells by DCF-DA assay, Hoechst 33342 staining, and MTT assay, respectively [39,48,51,52].

#### 3.3.3. Measurement of the Effect of Fucoidan on the Expression of Apoptosis-Related Proteins in UVB-Irradiated HaCaT Cells

The effect of fucoidan on the expression of the apoptosis-related proteins Bax, Bcl-xL, PARP, and cleaved Caspase-3 were assessed by Western blot assay. HaCaT cells were treated with fucoidan and irradiated with UVB, as described. After 24 h incubation, the cells were harvested and lysed. The protein level in each sample was measured by a BCA^TM^ kit. The Western blot protocol was performed according to the procedure, as described by Wijesinghe et al. [53].

### 3.4. Measurement of the Effect of Fucoidan on Melanogenesis

#### 3.4.1. Measurement of the Effect of Fucoidan on Mushroom Tyrosinase

The inhibitory effect of fucoidan on tyrosinase activity was measured according to the protocol described by Wang et al. [13]. Briefly, a reaction mixture (200 μL) containing phosphate buffer (50 mM, pH 6.5, 140 μL), l-tyrosine (1.5 mM, 40 μL), sample solution (10 μL), and mushroom tyrosinase solution (1000 units/mL, 10 μL) in a 96-well plate was reacted at 37 °C for 12 min. Then, the reaction was stopped by cooling the plate on ice for 5 min. The amount of dopachrome was measured at 490 nm using a microplate reader (BioTek, Synergy, UT, USA).

#### 3.4.2. B16F10 Cell Culture and Cytotoxicity Assay

The B16F10 melanoma cells (ATCC® CRL-6475™) were maintained in DMEM (containing 10% FBS and 1% P/S) and subcultured every 4 days. For the experiments, the cells were seeded at a density of 5 × 10^4^ cells/mL.

The toxicity of fucoidan to B16F10 cells was assessed by MTT assay. In brief, after seeding and incubating the B16F10 cells for 24 h, cells were treated with different concentrations of fucoidan (25, 50, and 100 µg/mL) for 72 h. The viability of the fucoidan-treated cells was then determined by MTT assay [49].

#### 3.4.3. Measurement of the Effect of Fucoidan on Intracellular Melanin Content of α-MSH-Stimulated B16F10 Cells

B16F10 cells were treated with fucoidan and stimulated with α-MSH (50 nM). After 72 h incubation, the α-MSH-stimulated B16F10 cells were harvested. The melanin content of α-MSH-stimulated B16F10 cells was assessed using the protocol described by Wang et al. [13].

#### 3.4.4. Measurement of the Effect of Fucoidan on Melanogenesis-Related Proteins in α-MSH-Stimulated B16F10 Cells

The effects of fucoidan on the expressions of melanogenesis-related proteins, including tyrosinase, TRP-1, TRP-2, MITF, and ERK–MAPK in α-MSH-stimulated B16F10 cells, were assessed by Western blot assay. The Western blot assay was performed according to the procedure described by Kim et al. [54].

### 3.5. Statistical Analysis

All experiments were conducted in triplicate. The data are expressed as the mean ± standard error (SE), and one-way ANOVA was used to compare the mean values of each treatment in SPSS 17.0. Significant differences between the means were identified by the Tukey test.

## 4. Conclusions

In this study, the anti-photoaging and anti-melanogenesis effects of fucoidan and the mechanisms involved were investigated. We found that fucoidan effectively protected HaCaT cells against UVB-induced photodamage by regulating apoptosis-related signaling pathways via intracellular ROS scavenging. In addition, fucoidan remarkably inhibited melanin biosynthesis in B16F10 cells by down-regulating melanogenesis-related proteins through ERK–MAPK pathway regulation. These results suggest that the fucoidan isolated from *H. fusiforme* possesses potent effects against skin photoaging and melanogenesis and could thus be considered for use as an ingredient in the pharmaceutical and cosmeceutical industries.
